# Breeding and Growth Performance of ‘Ningzhi 4’, a New Blackberry Cultivar with High Yield Potential and Good Quality in China

**DOI:** 10.3390/plants12081661

**Published:** 2023-04-15

**Authors:** Yaqiong Wu, Wenlong Wu, Chunhong Zhang, Lianfei Lyu, Weilin Li

**Affiliations:** 1 Jiangsu Key Laboratory for the Research and Utilization of Plant Resources, Institute of Botany, Jiangsu Province and Chinese Academy of Sciences (Nanjing Botanical Garden Mem. Sun Yat-Sen), Qian Hu Hou Cun No. 1, Nanjing 210014, China; ya_qiong@126.com (Y.W.); chzhang0714@163.com (C.Z.); njbglq@163.com (L.L.); 2 Co-Innovation Center for Sustainable Forestry in Southern China, Nanjing Forestry University, 159 Longpan Road, Nanjing 210037, China

**Keywords:** ‘Ningzhi 4’, blackberry (*Rubus* spp.), thornless, breeding, SSR

## Abstract

The thornless blackberry cultivar ‘Ningzhi 4’ was developed by the Institute of Botany, Jiangsu Province and the Chinese Academy of Sciences (Nanjing Botanical Garden Mem. Sun Yat-Sen). The new blackberry cultivar was selected from the ‘Kiowa’ (female parent) and ‘Hull Thornless’ (male parent) F1 hybrid. ‘Ningzhi 4’ had excellent plant characteristics, including thornlessness, semi-erect to erect canes, vigorous growth and good disease resistance. ‘Ningzhi 4’ had large fruit and high yield. In addition, the parents of the superior hybrid plant were further identified by SSR markers, which provided the basis for the fingerprint of the new blackberry cultivar ‘Ningzhi 4’. This is a commercial cultivar to be grown for fruit production for either shipping or local sales. It also has value as a home-garden plant. This unique type of blackberry fruit was a traditional summer fruit. This new cultivar has thornless semi-erect to erect canes and produces high-quality berries with large size, good firmness, excellent flavor, and potential for shipping and postharvest storage. The new blackberry cultivar ‘Ningzhi 4’ is adapted to all areas of southern China and is expected to replace or complement ‘Kiowa’, ‘Hull Thornless’, ‘Chester Thornless’ and ‘Triple Crown’. A local cultivar patent has been approved by the Jiangsu Variety Approval Committee as ‘*Rubus* spp. Ningzhi 4′ in 2020 (S-SV-RS-014-2020). In the future, ‘Ningzhi 4’ could be promoted as an advantageous thornless blackberry cultivar in the main production regions of China.

## 1. Introduction

*Rubus* L. plants have the characteristics of strong ecological adaptability, easy cultivation, fast forest formation, early yield and rich fruit nutrition [1,2]. All *Rubus* species have perennial roots and crowns. Blackberry (*Rubus* spp.) is a perennial shrubby fruit tree of *Rubus* in Rosaceae, which has been planted for more than 100 years [3]. Originating in North America, this plant was first introduced from Europe and America to China by our research team in 1986. The success or failure of a tree species introduction and the speed of its production effectiveness depends on the degree to which the ecological adaptability of the introduced object coincides with the habitat factors of the introduction site. Obviously, the ecological characteristics of blackberries coincide with the habitats in southeastern China. After more than 30 years of breeding and cultivation, blackberry has been widely used in China, with obvious income increase benefits for farmers [4]. Blackberry is the third generation of special fruit recognized by the Food and Agriculture Organization of the United Nations (FAO) and has received much attention due to its rich nutrition, medicinal, and economic value. According to the growth characteristics of the plant, it can be divided into four cultivation types: erect, semi-erect, trailing and semi-trailing [4]. Blackberry fruit is an aggregate berry consisting of a number of fleshy drupelets, each containing a single seed/pyrene around the central torus or receptacle. Blackberry fruit has a sweet and sour taste that is rich in nutrition. In addition to polysaccharides, organic acids and amino acids, it also contains vitamin C, pectin and mineral elements. It is a high-potassium and low-sodium fruit [4]. Blackberries also have unique healthcare functions [5]. For example, the total phenols in blackberries have antioxidant, heart protection, and lipid peroxidation-inhibiting effects [6]. The anthocyanins in blackberry fruits can prevent cardiovascular disease, diminish inflammation and enhance vision [6]. Mounting evidence suggests that eating berries can provide antioxidant and anticancer protection for humans and animals [7]. It is an important source of people’s intake of natural pigments, flavonoids, dietary fiber, vitamins, mineral elements and antioxidant substances [3,5,8]. With the development of the economy and the improvement of people’s living standards, the blackberry market has broad prospects. Therefore, cultivating some new and excellent varieties for planting and promotion will play a positive role in the large-scale production of blackberries.

‘Ningzhi 4’ was developed from a cross-breeding of ‘Kiowa’ (♀) × ‘Hull Thornless’ (♂) made during the blackberry flowering period in May 2006 at the Institute of Botany, Jiangsu Province and Chinese Academy of Sciences (Nanjing Botanical Garden Mem. Sun Yat-Sen), Nanjing, China. A total of 100 blackberry flowers were hybridized by emasculation and bagging, and 86 hybrid fruits and nearly 4000 hybrid seeds were obtained. The seed coat of blackberry was broken through concentrated sulfuric acid treatment, and the dormancy of seeds was broken through 2 months of wet sand storage and stratification. The seeds were sown in the early spring of 2007, and 168 hybrid seedlings were obtained. After one year of tending, the seedlings were planted in the breeding nursery in 2008. Through the investigation of growth, fruit quality and other character indices from 2008 to 2009, 6-6-3 plants (named ‘Ningzhi 4’) were selected in June 2009 from a population of 168 plants in a seedling field. During the ripening season, ‘Ningzhi 4’ was noted to have large fruit, good quality, and thornless and vigorous canes. In 2009, the plants were rapidly propagated by layering and tissue culture, and more than 200 clones were obtained. The parent ‘Hull Thornless’ is a vigorous, thornless, semi-erect type blackberry cultivar that is high yielding, with medium-sized and fairly soft fruit [4]. ‘Hull Thornless’ is widely promoted in China due to its good adaptability, growth potential and high yield. The maternal parent ‘Kiowa’ is a thorny, erect type of blackberry adapted to the moderate temperate of Arkansas and has prominent characteristics such as vigorous growth and large fruit. Thornlessness is a desired goal of most blackberry breeding programs, and thornlessness traits are recessive [9]. The thorn character of blackberry hybrid offspring is closely related to its parents. If one parent has thorns, the majority of subsequent generations (80–90%) have thorns. If both parents have no thorns, the offspring will also have a small number (3–5%) of plants with thorns [10]. The new cultivar ‘Ningzhi 4’ overcomes the female parent’s thorny character and maintains the male parent’s thornless character, which is beneficial to field cultivation and management.

False hybrids are often mixed in hybrid offspring. Accurate and rapid identification of true and false hybrids can improve the selection efficiency of breeding [11]. The traditional hybrid identification is generally conducted by morphological analysis. The identification cycle is long, and the results are easily affected by environmental factors such as climate, diseases and insect pests. Some characters with small differences are not easy to observe. At the molecular level, we can accurately and reliably identify the differences in the gene sequence. Simple sequence repeat (SSR) marker technology has the characteristics of accuracy, speed and convenience, providing an effective method for the identification of true and false hybrids [12]. As a second-generation molecular marker, it has been widely used in the identification of crop and forest varieties and the detection of seedling authenticity and purity [11,13,14]. In this study, *Rubus* SSR markers [15,16] were used to further test the genetic characteristics of ‘Ningzhi 4’, so as to provide a basis for determining the source of parents of superior plants and identifying the fingerprints of bred varieties.

**Figure 1:**
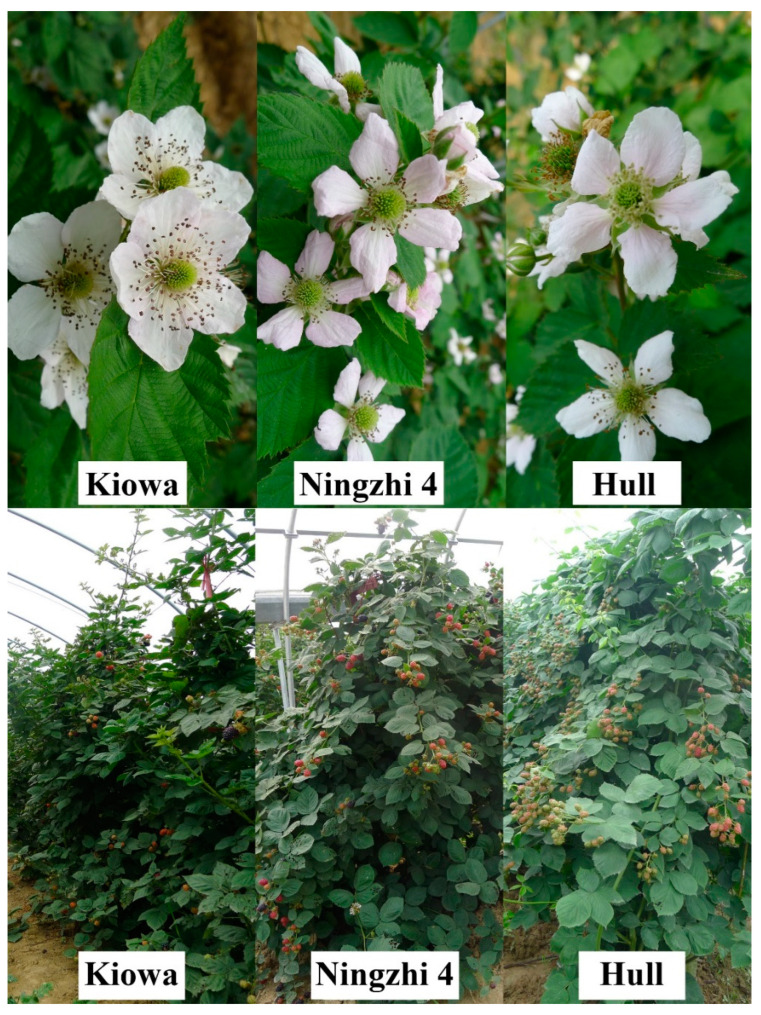
‘Kiowa’, ‘Ningzhi 4’ and ‘Hull thornless’ blackberry of flower morphology and fruiting.

The cross of elite thornless and large fruit selections results in the thornless plant and the enhancement of fruit size and quality in this cultivar. ‘Ningzhi 4’ combines the excellent characteristics of both parents, showing strong adaptability, large growth potential, high yield, large fruit and long fruit maturity (Figure 1). The fruits of ‘Ningzhi 4’ were shiny black, with good firmness and excellent flavor. It not only overcomes the bad character of the female parent ‘Kiowa’ with thorns but is also a thornless blackberry variety with large single fruit quality to date. Fruits were easily harvested, making them well suited to hand or machine harvesting. ‘Ningzhi 4’ is expected to perform well in areas where ‘Kiowa’, ‘Hull Thornless’, ‘Chester Thornless’ or ‘Triple Crown’ are adopted, including all areas of southern China. Furthermore, flat topography or sunny slope with slightly acidic soils (pH = 6.0~6.6), loam or sandy loam are suitable for cultivating ‘Ningzhi 4’.

## 2. Results and Discussions

### 2.1. Phenological Phase of Blooming and Fruit Ripening

The bloom date of ‘Kiowa’ was usually earlier than all comparison cultivars. ‘Ningzhi 4’ began to bloom 12 d later than ‘Kiowa’, 5 d before ‘Hull Thornless’ and 8 d before ‘Chester Thornless’ plants (Table 1). The date of 50 % bloom was 4–7 d before ‘Hull Thornless’ and ‘Chester Thornless’ but 13 d later than ‘Kiowa’. The mean date of the first ripe fruits on ‘Ningzhi 4’ was 17 June, which was only 3 d later than for ‘Kiowa’, 8 d before ‘Hull Thornless’ and 18 d before ‘Chester Thornless’. The peak and end ripening periods of ‘Ningzhi 4’ were earlier than those of the other three cultivars. The very early fruit provision should make this cultivar very attractive to homeowners and local marketers.

### 2.2. Fruit Characteristics

**Figure 2:**
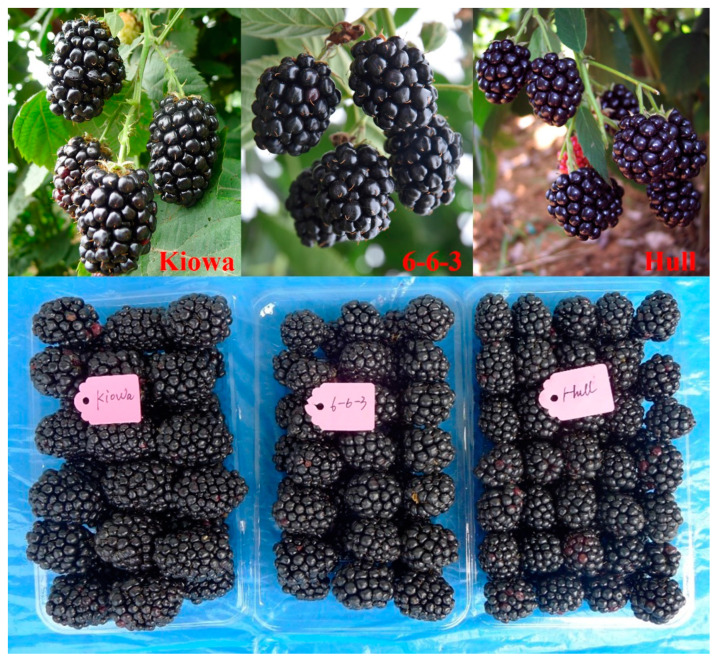
Ripe fruits of ‘Kiowa’, ‘Ningzhi 4’ (6-6-3) and ‘Hull Thornless’.

The size, shape index and color of the fruit are the key factors that determine the price of fresh fruit [17]. There is a positive correlation between fruit weight and fruit size [18]. In actual production, the flavor quality of the fruit is initially judged based on its size and shape. Therefore, the fruits are large dark purple, and the shiny blackberry is more attractive to consumers. The ripe fruit of ‘Ningzhi 4’ is attractive with glossy and shiny black color, and its elongated fruit shape is similar to ‘Kiowa’ (Table 1, Figure 2). Transverse and longitudinal diameters (width and length) of ‘Ningzhi 4’ were averaged 2.72 and 2.10 cm, larger than that of ‘Hull Thornless’ (2.45, 2.03 cm) and ‘Chester Thornless’ (2.40, 2.07 cm) but smaller than that of ‘Kiowa’ (3.58, 2.65 cm). ‘Ningzhi 4’ berries were heavier than those of ‘Hull Thornless’ and ‘Chester Thornless’ but lighter than those of ‘Kiowa’, whether at the beginning, peak or end of ripening (Table 1, Figure 2). Fruit firmness is an important index to evaluate the edible quality, storage performance and processing quality of fruit [18]. The fruit firmness of ‘Ningzhi 4’ was rated as 7.8, which is slightly higher than that of its parent cultivars ‘Kiowa’ and ‘Hull Thornless’, and slightly lower than the 8.0 rating for ‘Chester Thornless’ (Table 1), indicating consistent transportation potential. The flavor rating for ‘Ningzhi 4’ averaged 8.0, higher than that of ‘Kiowa’ (7.5) and ‘Chester (7.5) Thornless’, but lower than that of ‘Hull Thornless’ (8.5) (Table 1). The dry seed weight averaged 3.67 mg/seed for ‘Ningzhi 4’, slightly lighter than that of maternal ‘Kiowa’, but slightly heavier than that of paternal ‘Hull Thornless’.

### 2.3. Fruit Compositional Characteristics

The contents of soluble solids, acid and anthocyanin determine the nutritional value and taste of fruit [19]. The mature fruit of blackberry presents purplish red or purplish black, and its anthocyanin content determines the health value of the berry and the color of its processed products [17]. At the same time, changes in flavor substances such as sugar and acid also directly affect fruit quality [17]. The solid acid ratio is an important indicator of fruit sweetness, and its size is largely determined by the type and quantity of sugar contained in the fruit and the content of organic acids [20,21]. The taste of low-sugar and high-acid fruit is sour, which is not popular with most people, while the taste of low-acid and high-sugar fruit is light, which does not meet the requirements of fresh fruit [20]. Generally, the higher the solid-acid ratio is, the higher the sweetness of the fruit. The highest soluble solid concentration (SSC) was found in ‘Ningzhi 4’ (9.8%), whereas ‘Kiowa’ had the lowest SSC (8.1%) (Table 2). The total acid (TA) content of ‘Ningzhi 4’ fruit was 1.33%, near that of ‘Hull Thornless’ (1.30%), but lower than that of ‘Kiowa’ (1.48%) and ‘Chester Thornless’ (1.40%). The ratio of SSC to TA contents over the years in both Nanjing and Lishui indicated that berries were routinely sweeter than ‘Kiowa’ and ‘Chester Thornless’ berries.

Anthocyanins are the most studied bioactive substances in blackberries at present, and their content determines the color of the fruit [1]. The content of anthocyanins in blackberry fruits of different cultivars is mainly determined by their genotypes, and other factors (light, soil, tree age and harvest time) can also affect the content of anthocyanins in blackberry fruits [4,17]. The anthocyanin content averaged 80.65 mg/100 g for ‘Ningzhi 4’ fruit, which was close to ‘Kiowa’, higher than that of ‘Hull Thornless’ (75.46 mg/100 g) fruit, but lower than that of ‘Chester Thornless’ (106.68 mg/100 g). Therefore, this cultivar is recommended for functional component extraction potential.

### 2.4. Yield and Growth Habit

The main indicators for evaluating fruit trees include fruit quality, yield, fruit tree growth and adaptability. The yield of the Lishui test site was higher than that of Nanjing for all the cultivars. The production for ‘Ningzhi 4’ averaged 14.43 and 16.49 t/ha at Nanjing and Lishui, respectively, less productive than ‘Kiowa’ (15.12, 17.35 t/ha) but more productive than ‘Hull Thornless’ (12.96, 14.47 t/ha) and ‘Chester Thornless’ (13.07, 15.47 t/ha).

Canes of ‘Ningzhi 4’ were thornless and semi-erect to erect, similar to ‘Chester Thornless’ plants (Table 1). The ‘Ningzhi 4’ plants were observed to be very vigorous, and the vigor rating was close to ‘Kiowa’, higher than ‘Hull Thornless’ and ‘Chester Thornless’ (Table 1). The average health rating of ‘Ningzhi 4’ was the best among the four cultivars, near that of ‘Kiowa’ (Table 1). No anthracnose (*Colletotrichum* spp.) was found on berries or leaves of ‘Ningzhi 4’ after 5 years of planting. Moreover, this cultivar has very good field tolerance to root rot (*Phytophthora fragariae* var. *rubi*). The reaction of plants to rosette/double has not been determined definitively, but no symptoms have been observed on ‘Ningzhi 4’ after 5 years of planting. White drupelet development occurs during the ripening stage in some blackberry cultivars [22]. Very few to no white drupelets were found on ‘Ningzhi 4’. Additionally, uneven drupelet set has often been observed in ‘Navaho’ fruit because of some degree of sterility [4]. ‘Ningzhi 4’ has commendable fertility (data not shown) and a full drupelet set (Figure 2). In the present field tests, the cold hardiness of ‘Ningzhi 4’ was not fully evaluated, but no winter injury was found at a temperature of −10 °C (January 2011). No heat damage was found on plants or fruit up to 42 °C (August 2014).

### 2.5. Identification of the Hybrid Line ‘Ningzhi 4’

In recent years, the application of molecular marker technology in fruit tree germplasm identification, genetic relationship analysis, and early selection of breeding materials has developed rapidly [23,24]. SSR markers have the advantages of good repeatability, rich polymorphism, and strong reliability and have obvious advantages in identifying the purity and authenticity of fruit tree hybrids [25]. Because SSR markers are codominant and the genetic background of blackberry is relatively complex, 78 pairs of SSR molecular marker primers reported in the literature [15,16] were used to screen the two parents ‘Kiowa’ and ‘Hull Thornless’ of the hybrid line ‘Ningzhi 4’, of which 7 pairs of primers did not amplify clear bands, and the remaining 71 pairs of primers could amplify bands. There may be multiple DNA fragments produced by different primers, but only the main band with the largest amplification and the deepest staining is considered. The amplified fragment size of the main band was 70~500 bp, among which 11 pairs of primers had codominant differences between parents (Table S1), and the percentage of polymorphism was only approximately 15%. The SSR primers used in this study were developed from red raspberry and blackberry varieties in different raspberries [15,16]. Some of the amplified bands of F1 superior plants were dominant, and some were codominant, indicating that the applicability of SSR primers developed by raspberries under different genetic backgrounds is worth in-depth evaluation. To accurately identify the bred varieties through fingerprints, it was necessary to develop blackberry SSR primers in the future.

**Figure 3:**
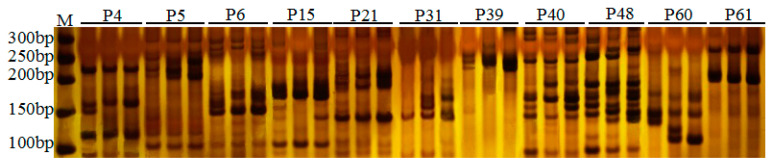
Amplified patterns of 11 pairs of polymorphic primers in ‘Ningzhi 4’ and its parents (‘Kiowa’ (♀) and ‘Hull Thornless’ (♂)). P4, P5, P6, P15, P21, P31, P39, P40, P48, P60 and P61 are 11 pairs of polymorphic primers, and the three channels are in the same group. The genomic DNA of the female parent ‘Kiowa’, male parent ‘Hull’ and ‘Ningzhi 4’ are amplified, respectively. M: 50 bp DNA ladder.

The amplification results of 11 pairs of polymorphic primers on the female parent ‘Kiowa’, male parent ‘Hull’ and excellent strain ‘Ningzhi 4’ showed that the proportion of polymorphic primers was 14.10%. The amplified bands of P4, P15, P21, P61 and other primers were the same as those of the male parent (Figure 3). P5, P6, P39, P40, P48 and other primers show the heterozygous type of both parents (Figure 3). P31 and P60 show the heterozygous type of both parents, but there are bands missing. Among the 11 pairs of polymorphic primers, 5 pairs of ‘Ningzhi 4’ were amplified as a two-parent heterozygous type, and some of them were partial to the male parent. It was speculated that its characteristics were between those of the parents or close to those of the male parent. SSR analysis technology will identify differences among different varieties at the DNA level, thereby scientifically and accurately determining the specificity, consistency, and stability of specific varieties [24,25]. SSR markers can provide objective, scientific, and accurate technical support for protecting breeding property rights, registering new varieties, and identifying and detecting the authenticity and purity of fruit tree varieties and seedlings.

## 3. Materials and Methods

### 3.1. Plant Growth Conditions

After selection, 2 test plots of ‘Ningzhi 4’ were established at Lishui Scientific Research Base (lat.: 31°35′14.50″ N, long. 119°09′41.24″ E, hereinafter referred to as “Lishui”) and Nanjing Botanical Garden (lat.: 32°03′17.29″ N and long. 118°49′44.69″ E, hereinafter referred to as “Nanjing”) of the Institute of Botany, Jiangsu Province and Chinese Academy of Sciences in 2009. The test design was a randomized block design. In Nanjing, 40 ‘Ningzhi 4’ plants were planted, and 20 plants of other cultivars were planted. In Lishui, 150 ‘Ningzhi 4’ plants (1.0 mu of land) were planted, and 50 plants of the other cultivars were planted. The planting row spacing was 2.50 m × 1.50 m, with optimal management conditions, sufficient fertilizer and water, and microirrigation conditions. The plants were obtained through rapid propagation by means of layering and tissue culture. Then, the regional tests were carried out to survey the plant growth and fruit outcome. In February 2011, ‘Ningzhi 4’ was planted with ‘Kiowa’, ‘Hull Thornless’ and ‘Chester Thornless’ (a popular cultivar at Lishui and Nanjing, used as a control) in the 2 test plots, respectively.

### 3.2. Soil and Planting Conditions

The plants were spaced 1.5 m apart in a row and 2.5 m apart. Trellises with wires at 1.8 and 2.0 m were used for all plants. The soil at Lishui is acidic clay loam with an organic matter content of 18.67 g/kg, a total nitrogen content of 1.25 g/kg, an available phosphorus content of 4.83 mg/kg and an available potassium content of 94.21 mg/kg. The soil in Nanjing is slightly acidic clay loam with an organic matter content of 11.25 g/kg, a total nitrogen content of 1.24 g/kg, an available phosphorus content of 7.54 mg/kg and an available potassium content of 62.21 mg/kg. In all plantings, standard cultural practices were used, including annual hand weeding, nitrogen fertilization (78 kg/ha N) using organic and inorganic compound fertilizers, normal irrigation through the drip system (typically 2.5–5.0 cm per week depending on rainfall), summer topping (the plants were topped at 0.8–1.0 m), the dead branches removal (after fruit harvest) and winter pruning (after defoliation and before sprouting) [26,27].

### 3.3. Main Methods of Plant Observational Data and Sensory Evaluation of Fresh Fruits

The observational data were collected at the 2 sites in 2014 and 2015. Ask 30 professionals engaged in the fruit tree industry to visually observe the plant growth traits (including vigor and health) and rate them on a scale of 1–10, of which 10 = erect, vigorous or thornless and 1 = trailing, poor growth or thorny. The fruit ratings included firmness (as measured subjectively by using finger pressure between the thumb and forefinger in the field on 8–10 berries, with a rating of 10 indicating very firm and 1 indicating extremely soft) and flavor (subjectively rated by tasting berries in the field, with a rating of 10 indicating the best). Data for 10% and 50% bloom, and first (5%), peak (50%), and last harvest (95%) dates were recorded for 2014 and 2015 and averaged for the replicated plots at Nanjing and Lishui.

### 3.4. Methods for Determination of Fruit Size and Quality

A total of 25-berry samples (from the upper, middle and lower layers of the blackberry plants) collected randomly once each season for 2 years were used to determine the fruit size and quality. The berry fruit size (transverse and longitudinal diameters) was measured by using a Vernier caliper (SMCT, Qualitot, Shanghai, China). The fruit shape index was used to calculate the ratio of the longitudinal diameter to the transverse diameter of blackberry fruit. The blackberry weight was calculated by the weighted mean through an electronic balance with a sensitivity of 10 mg. Seeds were extracted from the berries using a blender and dried at 70 °C for 24 h. Then, the 100-seed weight was measured. The data for fruit compositional characteristics (based on 25 berry samples randomly collected once each season for 2 years) included soluble solid, total acid and anthocyanin contents. The content of soluble solids was measured by the handheld refractometer (Atago, WYT-A, Tokyo, Japan). Use the automatic potentiometric titrator (Jinmai, ZD-2, Shanghai, China) to determine the total acid content. The content of anthocyanin in blackberry fruit was determined by the pH differential method using an ultraviolet spectrophotometer (Jinghua, 759, Shanghai, China). The data for yield were calculated by the actual yield per plant × the average area per plant during fruit ripening in 2015. These trials consisted of 4 replications.

### 3.5. DNA Extraction and SSR Primer Synthesis

At the beginning of May, 4–6 young leaves from the top down on the annual branches of 3 blackberry cultivars were collected. After collection, wipe off the dust on the surface of the leaves, place them in liquid nitrogen for rapid storage, and then place them in an—80 ultra-low temperature refrigerator for subsequent DNA extraction. The leaves of ‘Ningzhi 4’, ‘Kiowa’ and ‘Hull Thornless’ plants were collected to extract genomic DNA using a cetyltrimethylammonium bromide Plant Genomic DNA Kit (Zoman, Beijing, China). The DNA concentrations were estimated with a NanoDrop-1000 spectrophotometer (NanoDrop Technologies, Wilmington, DE, USA). DNA quality was assessed for each DNA sample on 1% agarose gel. A total of 78 pairs of SSR primers were used, including 4 pairs from the red raspberry cultivar ‘Meeker’, 8 pairs from the blackberry hybrid ‘Marion’ [16], and the remaining 66 pairs from the red raspberry cultivar ‘Glen Moy’ [15]. The primer synthesis was completed by Shanghai Invitrogen Biotechnology Co., Ltd.

### 3.6. SSR-PCR Reaction and Electrophoresis Analysis

PCR was carried out in a final volume of 10 μL containing: 1 μL 10 × buffer, 0.2 μL 2.5 mmol/L dNTPs, 2 μL 5 pmol/μL primer, 0.1 μL 5 U/μL Taq enzyme, 1 μL DNA template, and ddH_2_O 5.7 μL. The PCR conditions were as follows: 94 °C for 5 min followed by 35 cycles of 94 °C for 30 s, 45 °C for 30 s, 72 °C for 30 s and final elongation at 72 °C for 10 min. PCR amplification for all SSR markers was carried out using an ABI Veriti 96 PCR system (Thermo Fisher Scientific, MA, USA). The PCR products were resolved on an 8% nondenatured polyacrylamide gel (PAGE) and stained using a silver-staining protocol.

### 3.7. Statistical Analysis of Data

The statistical analysis of all experimental data was performed using SPSS 22.0 software (SPSS, Chicago, IL, USA) by LSD and Duncan’s multiple range test (*p* ≤ 0.05).

## 4. Conclusions

To develop blackberry cultivars suitable for local use and with independent intellectual property rights, it is particularly necessary to conduct cross-breeding research using domestic and foreign resources. ‘Ningzhi 4’ had excellent plant characteristics. This cultivar is a thornless shrub with vigorous growth, mature black fruit, good flavor and sweet taste. Its yield is higher than that of the ‘Hull’ and ‘Chester’ cultivars. Fruits were easily harvested, making them well suited to hand or machine harvesting. ‘Ningzhi 4’ is expected to perform well in areas where the ‘Kiowa’, ‘Hull Thornless’, ‘Chester Thornless’ or ‘Triple Crown’ cultivars are adapted. ‘Ningzhi 4’ should be planted in slightly acidic soil, loam or sandy loam, which can be popularized in most provinces of the Yangtze River basin in China, such as Jiangsu, Anhui, Zhejiang, and Hubei provinces.

## Figures and Tables

**Table 1:** Growth habit, phenological phase and fruit characteristics of four blackberry cultivars at Nanjing and/or Lishui in 2014–2015.

Characteristic	Cultivar			
	‘Ningzhi 4’	‘Kiowa’	‘Hull Thornless’	‘Chester Thornless’
Plant ^z^				
Vigor Health Erectness Thorniness	8.5 ± 0.5 8.8 ± 0.5 8.0 ± 0.8 -	8.8 ± 0.5 8.5 ± 0.6 7.5 ± 0.6 +	7.5 ± 0.6 7.5 ± 1.0 7.0 ± 0.5 -	8.0 ± 0.6 8.3 ± 0.5 8.0 ± 0.6 -
Bloom date ^y^				
10% bloom 50% bloom	10 May 18 May	28 Apr. 5 May	15 May 22 May	18 May 25 May
Ripening date ^y^				
Beginning Peak End	17 June 2 July 22 July	14 June 26 June 5 Aug.	25 June 7 July 25 July	5 July 22 July 10 Aug.
Berry ripe rating ^z,y^				
Beginning	7.5 ± 0.5	7.5 ± 1.2	7.0 ± 1.0	5.3 ± 0.5
Berry size (cm) ^y^				
Length Width Fruit shape index	2.72 b ^x^ 2.10 a 1.30 bc	3.58 c 2.65 b 1.35 c	2.45 a 2.03 a 1.21 ab	2.40 a 2.07 a 1.16 a
Single berry weight (g) ^y^				
Beginning Peak End	8.8 b ^x^ 7.6 b 6.5 c	15.2 c 12.8 c 7.6 d	7.1 a 5.6 a 5.1 b	7.0 a 5.2 a 4.6 a
Fruit quality ^z,y^				
Firmness Flavor	7.8 ± 0.6 8.0 ± 0.5	7.5 ± 0.5 7.5 ± 0.6	7.5 ± 0.6 8.5 ± 0.5	8.0 ± 0.5 7.5 ± 0.4
Seed dry weight (mg/seed) ^w^	3.67 c ^x^	3.83 d	3.51 b	3.16 a

^z^ Rating scale of 1–10; 10 = best, early ripe, vigorous, strong resistance or erect; 1 = poor, late ripe, poor growth, poor resistance or trailing; ^y^ Means of two years data collected from only Nanjing; ^x^ Mean separation within columns by LSD and Duncan’s multiple range test, *p* ≤ 0.05; Lines with different lowercase letters significantly (*p* < 0.05) differed based on Duncan’s multiple range test; ^w^ Average seed weight of from a 1000-seed sample collected from mature fruit in 2015; +: thorny; -thornless.

**Table 2:** Soluble solid content (SSC), total acid (TA) content and anthocyanin content of four blackberry cultivars in Nanjing in 2014–2015.

Characteristic ^z^	Cultivar			
	‘Ningzhi 4’	‘Kiowa’	‘Hull Thornless’	‘Chester Thornless’
Soluble solids (%)	9.80 d ^y^	8.10 a	9.50 c	8.70 b
Total acids (%)	1.33 b	1.48 d	1.30 a	1.4 c
Ratio SSC: TA	7.37 c	5.47 a	7.31 c	6.17 b
Anthocyanin content (mg/100 g)	80.65 b	80.76 b	75.46 a	106.68 c

^z^ Means of two years data (2014 and 2015); ^y^ Mean separation within columns by LSD and Duncan’s multiple range test, *p* ≤ 0.05. Lines with different lowercase letters significantly (*p* ≤ 0.05) differed based on Duncan’s multiple range test.

## Data Availability

In December 2020, ‘Ningzhi 4’ passed the expert identification of Jiangsu Provincial Forest Variety Approval Committee, and the Improved Variety number is S-SV-RS-014-2020. A list of nurseries licensed to propagate and sell ‘Ningzhi 4’ can be obtained from Wenlong Wu, Research Center for Pomology, Institute of Botany, Jiangsu Province and Chinese Academy of Sciences, Nanjing, Jiangsu, China. All data and materials supporting the conclusions of this study are included in the article.

## Supplementary Materials

The following supporting information can be downloaded at: https://www.mdpi.com/article/10.3390/plants12081661/s1. Table S1. Polymorphic primers in parent cultivars of blackberry ‘Ningzhi 4’.
