# Supplementary material for: Breeding and Growth Performance of ‘Ningzhi 4’, a New Blackberry Cultivar with High Yield Potential and Good Quality in China
Source: Plants (Basel). 2023 Apr 15;12(8):1661. doi: 10.3390/plants12081661 (PMC10147019; doi:10.3390/plants12081661)
Supplement: Supplementary file 1 [file plants-12-01661-s001.zip › plants-2281274-supplementary.pdf]

Table S1. Polymorphic primers in parent cultivars of blackberry 'Ningzhi 4'

Number	Name	Repeat motifs	Primers 5'-3' (Forward/Reverse)
P4	Rubus6a	(ct)16(ca)32	TGCATGTGACTTTGCATCTCT/ GCACTGAAAAATCATGCATCTG
P5	Rubus12a	(ct)7(at)6(gt)10	ATTCCCCGCCTCAGAATAAT/AAGGTTTGTGACGGGAACAG
P6	Rubus16a	(at)8(gt)11	TGTTGTACGTGTTGGGCTTT/ GGGTGTTTGCCAGTTTCAGT
P15	Rubus45c	(t)10-(a)11-(ga)15	GAGGGGCAATTAAAGGGTTT/TGTTGTAATTTGGTTTATCCTTGG
P21	Rubusr76b	(ct)5-(ct)4	CTCACCCGAAATGTTCAACC/ GGCTAGGCCGAATGACTACA
P31	Rubus123a	(ag)8	CAGCAGCTAGCATTTTACTGGA/ GCACTCTCCACCCATTTCAT
P39	Rubus166b	(tc)15	CCGCAAGGGTTGTATCCTAA/ GCATGAGGGCGATATAAAGG
P40	Rubus167a	(tc)9	AACCCTAAGCCAAGGACCAT/ CACCACCCATGACAGTCAGA
P48	Rubus251a	(ga)10	GCATCAGCCATTGAATTTCC/CCCACCTCCATTACCAACTC
P60	Rubus275a	(ag)27	CACAACCAGTCCCGAGAAAT/ CATTTCATCCAAATGCAACC
P61	Rubus277a	(a)11(ag)8	GCCCCATCCTGTACAAAGAA/ TTGCAACAAAGGTACGTAATGG
